# Extended flow cytometry characterization of normal bone marrow progenitor cells by simultaneous detection of aldehyde dehydrogenase and early hematopoietic antigens: implication for erythroid differentiation studies

**DOI:** 10.1186/1472-6793-8-13

**Published:** 2008-05-29

**Authors:** Peppino Mirabelli, Rosa Di Noto, Catia Lo Pardo, Paolo Morabito, Giovanna Abate, Marisa Gorrese, Maddalena Raia, Caterina Pascariello, Giulia Scalia, Marica Gemei, Elisabetta Mariotti, Luigi Del Vecchio

**Affiliations:** 1 Servizio di Immunoematologia e Medicina Trasfusionale, Ospedale A. Cardarelli, Napoli, Italy; 2 Dipartimento di Biochimica e Biotecnologie Mediche (DBBM) – CEINGE – Biotecnologie Avanzate, Napoli, Italy

## Abstract

**Background:**

Aldehyde dehydrogenase (ALDH) is a cytosolic enzyme highly expressed in hematopoietic precursors from cord blood and granulocyte-colony stimulating factor mobilized peripheral blood, as well as in bone marrow from patients with acute myeloblastic leukemia. As regards human normal bone marrow, detailed characterization of ALDH^+ ^cells has been addressed by one single study (Gentry *et al*, 2007). The goal of our work was to provide new information about the dissection of normal bone marrow progenitor cells based upon the simultaneous detection by flow cytometry of ALDH and early hematopoietic antigens, with particular attention to the expression of ALDH on erythroid precursors. To this aim, we used three kinds of approach: i) multidimensional analytical flow cytometry, detecting ALDH and early hematopoietic antigens in normal bone marrow; ii) fluorescence activated cell sorting of distinct subpopulations of progenitor cells, followed by *in vitro *induction of erythroid differentiation; iii) detection of ALDH^+ ^cellular subsets in bone marrow from pure red cell aplasia patients.

**Results:**

In normal bone marrow, we identified three populations of cells, namely ALDH^+^CD34^+^, ALDH^-^CD34^+ ^and ALDH^+^CD34^- ^(median percentages were 0.52, 0.53 and 0.57, respectively). As compared to ALDH^-^CD34^+ ^cells, ALDH^+^CD34^+ ^cells expressed the phenotypic profile of primitive hematopoietic progenitor cells, with brighter expression of CD117 and CD133, accompanied by lower display of CD38 and CD45RA. Of interest, ALDH^+^CD34^- ^population disclosed a straightforward erythroid commitment, on the basis of three orders of evidences. First of all, ALDH^+^CD34^- ^cells showed a CD71^bright^, CD105^+^, CD45^- ^phenotype. Secondly, induction of differentiation experiments evidenced a clear-cut expression of glycophorin A (CD235a). Finally, ALDH^+^CD34^- ^precursors were not detectable in patients with pure red cell aplasia (PRCA).

**Conclusion:**

Our study, comparing surface antigen expression of ALDH^+^/CD34^+^, ALDH^-^/CD34^+ ^and ALDH^+^/CD34^- ^progenitor cell subsets in human bone marrow, clearly indicated that ALDH^+^CD34^- ^cells are mainly committed towards erythropoiesis. To the best of our knowledge this finding is new and could be useful for basic studies about normal erythropoietic differentiation as well as for enabling the employment of ALDH as a red cell marker in polychromatic flow cytometry characterization of bone marrow from patients with aplastic anemia and myelodysplasia.

## Background

Aldehyde dehydrogenase (ALDH) is a family of enzymes involved in metabolism of aldehydes to their corresponding carboxylic acids [1]. It plays an important role in metabolism of vitamin A as well as in mechanisms of resistance to alchylating agents, e.g. cyclophosphamide [2]. For these reasons, ALDH is considered a protecting or detoxifying enzyme, able to preserve stem cells from cytotoxic effects [2-4]. One of the accepted technologies to identify human hematopoietic stem cells (HSC) is based upon flow cytometry (FCM) detection of ALDH enzymatic activity [2]. In particular, Storms *et al *[2] designed a substrate for ALDH, termed BODIPY aminoacetaldehyde (BAAA), which consists of an aminoacetaldehyde moiety bonded to the BODIPY fluorochrome. Once BAAA diffuses freely into cells and it is converted by ALDH into BODIPY aminoacetate (BAA), it remains trapped intracellularly, so emitting green fluorescence [2].

Recently, the functional role of ALDH has been elucidated in a study, in which the specific inhibitor of ALDH, diethylaminobenzaldehyde (DEAB), was able to alter the molecular and cellular mechanisms that control self-renewal capacity of human HSC [5].

The evidence of ALDH involvement in the physiology of HSC was further highlighted by a series of studies devoted to purification/analysis of highly immature progenitor cells, particularly in human cord blood (CB) as well as in murine bone marrow (BM) [6-8]. The importance of ALDH in human hematopoiesis was also testified by a recent study in which the Authors tried to purify HSCs by combining FCM cell sorting and Hoechst-33342 efflux ability (the so called "Side Population") [9]. At variance with previous findings obtained in mouse, human BM hematopoietic cells able to exclude Hoechst-33342 did not correspond to highly immature HSCs. On the other hand, the Authors proposed that ALDH activity had to be considered as the reference method for the detection of immature HSCs in human BM, at the same time emphasizing the need of studies about expression pattern of ALDH in comparison with other hematopoietic cell markers in this tissue [9].

Thanks to the relevant advances deriving from studies performed by Gentry and coworkers [10,11], it was definitely understood that ALDH^bright ^cell population in banked cord blood is highly enriched in hematopoietic colony forming cells (CFC-H), compared to ALDH^dim ^cell populations [10]. Moreover, cell sorting of ALDH^bright ^cells in human BM enables a marked enrichment of CFC-H as well as of endothelial and fibroblast colonies [11].

Starting from these literature data, we carried out a comprehensive FCM characterization of human normal BM by detecting ALDH in combination with other hematopoietic cell markers. To this aim, we used a multidimensional polychromatic approach, according to international guidelines [12].

The goal of our work was to better define the expression of surface antigens classically associated with early steps of hematopoiesis such as CD38, CD45RA, CD71, CD105, CD117 and CD133 in three BM cellular compartments identified on the basis of ALDH and CD34 expression. We were also interested to derive information about the possible erythroid lineage commitment of these identified subpopulations of cells.

## Methods

Normal BM samples were obtained, following informed consent, from 23 patients with non-Hodgkin lymphoma (age 20–81, median 45) who underwent BM aspiration in the context of routine clinical practice. All samples showed complete absence of neoplastic cells when analyzed by smear morphology, BM biopsy and FCM. Peripheral blood counts were in normal ranges. Thus, all BM aspirates included in the study were operationally considered as normal. Written informed consent was obtained from all patients before the study.

Three patients with pure red cell aplasia (PRCA, age 36–40) were also included in the study. Also in this case, BM samples were obtained following informed consent.

In the first phase of the study, we analyzed BM cells by four-color FCM, detecting ALDH activity along with the expression of a series of hematopoietic antigens. Directly conjugated fluorescent antibodies were used reacting with the following antigens: CD34, CD38, CD45, CD45RA, CD71, CD105, CD117, CD133 and CD235a. In particular, PE conjugated monoclonal antibodies (MoAb) against CD133, CD117, CD45RA and CD105 were obtained from Miltenyi Biotec (Bergisch Gladbach, Germany), Caltag (Burlingame, CA, USA), Beckman-Coulter (Miami, FL, USA) and R&D Systems (Minneapolis, MN, USA), respectively. PE-conjugated anti-CD38, anti-CD34 and anti-CD235a, along with APC-conjugated anti-CD71, PE-Cy7 conjugated anti CD34 and PerCP-conjugated anti-CD45 were purchased from Becton Dickinson (San Jose, CA, USA).

For all antibody staining experiments, 50 μl of whole BM sample were incubated at 4°C for 30 min in the presence of appropriate amount of MoAbs in PBS. Successively, the mixtures were diluted 1:20 in ammonium chloride (NH_4_Cl) lysing solution, then incubated at room temperature for 5 minutes and finally washed with staining media prior to flow cytometry analysis. In experiments where BM samples were stained with BAAA and additional antibodies, the antibody reagents were incubated with the cells at a concentration of 10^6 ^cells/ml for 15 minutes prior to BAAA staining. Cells with low side scatter and high levels of BAAA staining were defined as "Side Scatter Low ALDH Bright" (SSC^lo^ALDH^br^) cells. ALDH activity was assessed by the BODIPY-based ALDEFLUOR assay (Becton Dickinson), according to the manufacturer method. As negative control, we used DEAB, a potent inhibitor of ALDH activity. Antibody and BAAA stained cells were analyzed on a FACSCalibur flow cytometer (Becton Dickinson) equipped with a 488-nm argon laser and a 635-nm red diode laser. BODIPY fluorescence was excited at 488 nm and fluorescence emission was detected using a standard fluorescein isothiocyanate (FITC) 530/30 bandpass filter. Levels of CD antigen expression were displayed as mean fluorescence intensity (MFI). The software used for cytometric analysis was Paint-a-gate (Becton Dickinson).

Cell sorting experiments were performed by the cell sorter FACSAria (Becton Dickinson) equipped with blue, red, and violet lasers. Dead cells were excluded by analyzing forward scatter (FSC) *vs *side scatter (SSC) dot plots. Doublets were excluded by FSC-H *vs *FSC-A dot plots. Three populations of cells were sorted: ALDH^-^CD34^+^, ALDH^+^CD34^+ ^and ALDH^+^CD34^-^. In all experiments, purity was higher than 95% of desired cells.

Immunophenotyping of patients with PRCA was performed by the flow cytometer FACSCanto (Becton Dickinson), by using a 5-colour strategy (1^st ^channel: ALDH; 2^nd ^channel: CD235a-PE; 3^rd ^channel: CD45-PerCP; 4^th ^channel: CD71-APC; 5^th ^channel: CD34-PE-Cy7).

Induction of differentiation studies were performed according to Malik et al (13). In brief, ALDH^+^CD34^+ ^and ALDH^+^CD34^- ^cells were purified from three different normal bone marrow samples and were cultured at a density of 10^5 ^cells/mL in Iscove's Modified Dulbecco's Medium (IMDM; GIBCO, Grand Island, NY), 1% deionized bovine serum albumin (BSA; Sigma, St Louis, MO), 10^-4 ^mol/L 2-mercaptoethanol, 10^-6 ^mol/L hydrocortisone, 100 U/mL penicillin-streptomycin, and 2 mmol/L L-glutamine with the following recombinant cytokines: 10 U/mL of recombinant human (rH) Epo (Amgen, Thousand Oaks, CA), 0.001 ng/mL rH granulocyte-macrophage colonystimulating factor (GM-CSF), and 0.01 U/mL rH interleukin-3 (IL-3; Immunex Corp, Seattle, WA) and incubated in 5% CO2 at 37°C. We strictly followed the indications of the reported method [13], culturing ALDH^+^CD34^+ ^cells and ALDH^+^CD34^- ^cells in the aforementioned culture conditions for 4 days in order to demonstrate our hypothesis that ALDH^+^CD34- cells were committed erythroid precursors.

Descriptive statistics included medians, lowest and highest values, and 95% CI for the medians. Statistical significance was assessed by paired Wilcoxon test.

## Results

**Figure 1:**
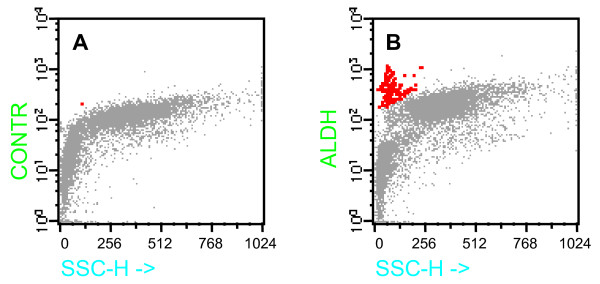
**Flow cytometric analysis of BM cells of a representative patient.** Panel B shows BM cells stained with BAAA. Panel A shows BM cells stained with BAAA and the ALDH inhibitor, DEAB. Red color indicates the SSC^lo^ALDH^br ^population. Grey color indicates the entire population of nucleated cells following to NH_4_Cl lysis.

Intracellular fluorescence staining of ALDH was combined with SSC with the aim of facilitating the identification of ALDH^+ ^stem cells and progenitor cells. In facts, it is well known that HSCs and progenitors typically have low SSC on FACS analysis [14]. A population of cells with low SSC and high levels of BAAA staining (SSC^lo^ALDH^br^) was evident in BM aspirates of all the 23 subjects studied (Fig 1, panel B). In contrast, when BM aspirate was stained simultaneously with BAAA and DEAB, a potent inhibitor of ALDH, no SSC^lo^ALDH^br ^cells were noted (Fig 1, panel A). The absence of ALDH staining in the presence of ALDH inhibitor confirms that the presence of SSC^lo^ALDH^br ^population is specifically due to high-level ALDH activity in these cells.

**Figure 2:**
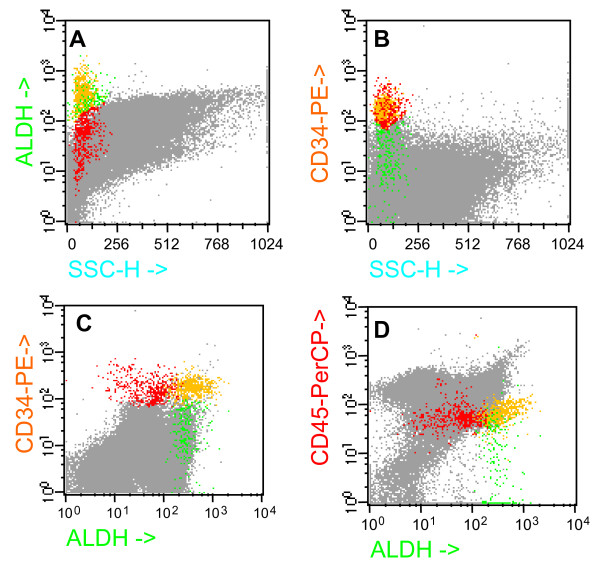
**Multidimensional flow cytometry analysis of BM cells according to the simultaneous detection of ALDH, CD34 and CD45.** Green cell population: ALDH+CD34- cells; red cell population: ALDH-CD34+ cells; yellow cell population: ALDH+CD34+ cells; grey cell population: the entire population of nucleated cells following to NH_4_Cl lysis. Note that ALDH+CD34- cells are CD45 negative.

In order to better define the BM cellular compartments in terms of ALDH and CD34 expression, we performed a multimensional FCM analysis using the Paint-a-gate software (Becton Dickinson). We identified three distinct populations of cells in all 23 samples analyzed: ALDH^+^/CD34^-^, ALDH^-^/CD34^+ ^and ALDH^+^/CD34^+^. In particular, in figure 2 (panel A) we first imposed a green gate on ALDH^+ ^cells, then a red gate on CD34^+ ^cells (panel B) and finally the events falling at the same time in the green as well as red gates were automatically depicted in yellow by the software. The median percentages of ALDH^+^/CD34^- ^(green), ALDH^-^/CD34^+ ^(red) and ALDH^+^/CD34^+ ^(yellow) cells were 0.57% (95% CI for the median 0.40 to 0.76), 0.53% (0.40 to 1.09) and 0.52% (0.24 to 0.68), respectively. The denominator of these percentages was composed by the entire population of nucleated bone marrow cells following to NH_4_Cl lysis of mature erythrocytes. Note that the green population (ALDH^+^CD34^-^) was simultaneously negative for CD34 and CD45 (Fig 2, panels C and D, respectively).

Starting from this point, the work was carried out with the aim of identifying the immunophenotype of the three aforementioned cell populations.

Firstly, we focused on differential expression of CD38, CD45RA, CD117 and CD133 in CD34^+^ALDH^+ ^and CD34^+^ALDH^- ^cells (Table 1). As compared to CD34^+^ALDH^- ^cells, CD34^+^ALDH^+ ^cells were characterized by lower expression of activation-differentiation antigens CD38 (median values 154 *vs *253, p < 0.01) and CD45RA (39 *vs *140, p < 0.01) and by higher expression of stem cell antigen CD117 (43 *vs *23, p < 0.01) and CD133 (15 *vs *9, p < 0.01).

**Table 1:** Expression of surface antigens on CD34+ALDH+ and CD34+ALDH- progenitor cells.

	*CD34+ ALDH+*			*CD34+ ALDH-*			
Antigen	Median	Range	95% CI	Median	Range	95% CI	p
CD38	154*	31–267	108–200	253	167–518	224–318	<0.01
CD45RA	39	14–174	28–69	140	32–258	94–168	<0.01
CD117	43	22–67	34–46	23	5–42	19–33	<0.01
CD133	15	12–28	13–27	9	6–30	6–16	<0.01

* Values are expressed as mean fluorescence intensity (MFI). Negative controls on CD34+ gated cells showed in any cases MFI lower than 5 units.

**Figure 3:**
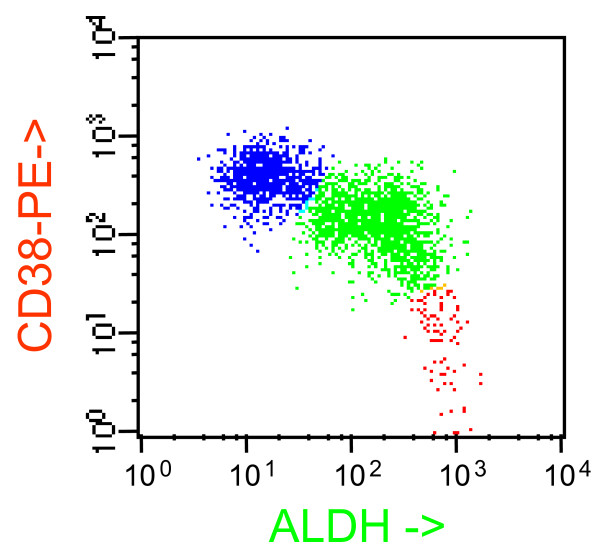
**Analysis of CD34+ gated cells shows that CD38 expression is inversely correlated with ALDH positivity.** Cells with brightest expression of ALDH are completely negative for CD38 (red cells). This subset corresponds to very early CD34+ cells. As cell differentiation proceeds, cells lose ALDH and acquire CD38 (blue cells). Green cells represent an intermediate stage.

Taken together, ALDH, CD133 and CD117 compose an immunophenotypic mosaic related to an undifferentiated status, while CD38 and CD45RA expression indicates a higher degree of cell maturation. To confirm this view, figure 3 shows the inverse correlation between CD38 and ALDH. Interestingly, in all cases studied the highest expression of ALDH strictly corresponded to the lowest expression of CD38.

**Figure 4:**
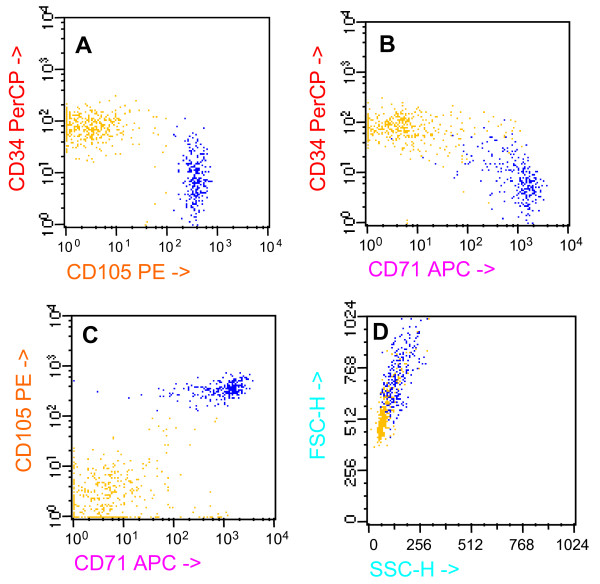
**Demonstration that ALDH+CD34- cells are early erythroid cells.** Blue: ALDH+CD34- cells. Yellow: ALDH+CD34+ cells. Cells were stained with ALDEFLUOR, CD105-PE, CD34-PerCP and CD71-APC. Gate was imposed on ALDH+ cells. In panel A, all CD34- cells are CD105+. In panel B, all CD34- cells are CD71^bright^. In panel C, CD105 and CD71 are completely co-expressed. Panel D shows that erythroid cells are larger than CD34+ progenitor cells. This case is one representative example of a phenomenon observed in all BM specimens.

Our attention was focused on ALDH^+^/CD34^- ^cell population. As shown in Figure 4, ALDH^+^/CD34^- ^cells (depicted in blue) were characterized by CD105 bright expression (panel A) accompanied by CD71 high expression level (panel B). Moreover, CD105 and CD71 antigens were tightly co-expressed (Fig. 4, panel C) and overall population defined by ALDH, CD105 and CD71 positivity was also characterized by high volume, as detected by forward scatter measurement (FSC, Fig. 4, panel D).

**Figure 5:**
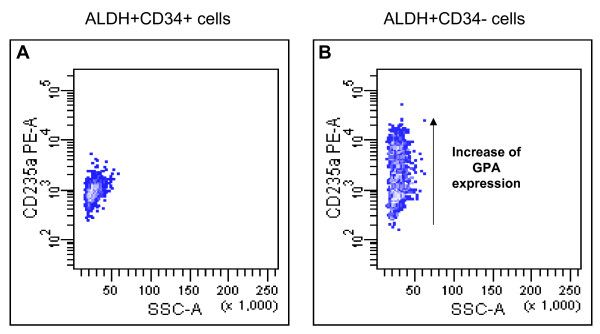
**Demonstration that ALDH+CD34- cells are committed toward erythropoiesis.** Panel A: sorted ALDH+CD34+ cell fraction, induced to differentiate for 4 days in the presence of erythropoietin, GM-CSF and IL3. Panel B: sorted ALDH+CD34- cell fraction, induced to differentiate in the same conditions. These results represent a typical experiment of the 3 performed.

Figure 5 shows in a representative case (we studied three normal BM samples) that isolated ALDH^+^CD34^- ^cells are committed towards erythropoiesis, since they can be easily induced to express GPA/CD235a by a brief cell culture (4 days) with a medium containing erythropoietin, GM-CSF and IL3. As shown in the figure, while ALDH^+^CD34^+ ^cells (panel A) remained GPA-negative following the exposure to the aforementioned agents, ALDH^+^CD34^- ^cells (panel B) were clearly induced to differentiate into GPA-positive cells.

**Figure 6:**
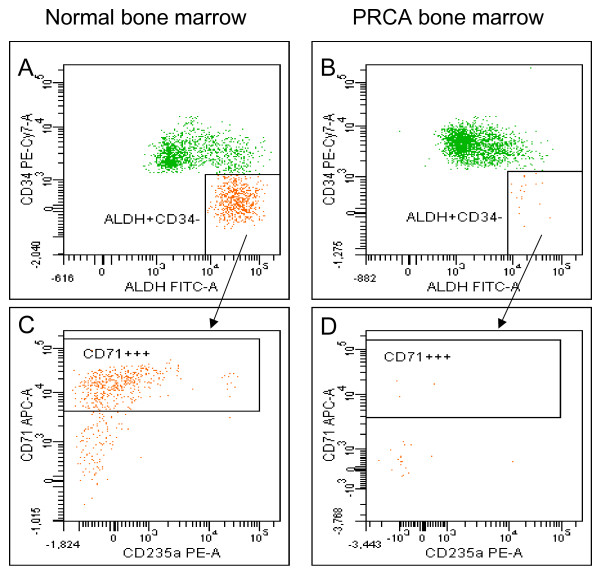
**Demonstration that PRCA patients are devoid of ALDH+CD34- cell population.** Panel A: expression of ALDH and CD34 in normal bone marrow. Panel B: expression of ALDH and CD34 in PRCA bone marrow (note the striking depletion of ALDH+CD34- cell population). Panel C: CD71 expression on ALDH+CD34- cells in human normal bone marrow. Panel D: absence of CD71 expression in PRCA bone marrow.

Figure 6 is the final demonstration, obtained in a disease model, that ALDH^+^CD34^- ^cells belong to an early erythroid subpopulation. In facts, PRCA patients are completely deprived of this cell subpopulation. Figure 6 shows one representative experiment (we studied three patients) in which normal bone marrow contains a population of ALDH^+^CD34^- ^cells (panel A), which is also CD71^bright ^(panel C). By contrast, PRCA bone marrow is almost completely deprived of ALDH^+^CD34^-^CD71^bright ^cells (panel B and D).

## Discussion

In spite of the existence of a series of studies describing ALDH expression in human CB [4,6,8,10], peripheral blood stem cells [3] and even acute myeloid leukemia (AML) BM cells [1] data aiming to characterize normal BM ALDH^+ ^cells are still limited to a few recent reports [9,11].

The use of NH_4_Cl lysed, unseparated bone marrow for immunophenotypic studies is strongly recommended by clinico-hematological guidelines [12,15]. In this work we decided to follow these recommendations, so that BM samples were not processed by density gradient separation to the aim of avoiding unwanted selection of cell subsets, and, at the same time, of not interfering with assay interpretation [12].

Our interest in ALDH detection relies on the consideration that FCM based ALDH activity assessment is exploited in order to evidence a conserved stem cell function, rather than to merely identify a stem cell antigen [16].

The first result of this study, based upon the simultaneous identification of ALDH activity and a detailed immunophenotype profile, is that the contemporary expression of CD34 and high levels of ALDH is able to identify primitive BM hematopoietic progenitors, clearly expressing high amounts of CD117 and CD133 as well as low amounts of CD38 and CD45RA. These data are in keeping with findings reported by Hess, Pearce and Gentry groups on CB cells [4,9,10]. They found that ALDH^bright ^cells were able to give multilineage hematopoietic repopulation and, when compared to their ALDH^lo ^counterparts, they expressed higher amounts of CD133, CD31, CD117 and lower amounts of CD38 [4,9,10].

Our study shares some arguments with the work by Gentry *et al *on human bone marrow [11]. Similarly to our work, these Authors characterized ALDH^bright ^cells with a series of MoAbs directed to surface antigens, e.g. CD34, CD117, CD133, CD105, CD166, CD127, CD38, HLA-DR and CD45. Nevertheless, there is one difference between our study and Gentry's work. While Gentry and coworkers [11] used a 3-color plus 7-AAD (7-amino-actinomycin D) scheme, thus comparing the expression of ALDH with 2 surface antigens, we combined ALDH analysis with 3 surface antigens. Such a multidimensional strategy allowed us to obtain the core finding of our work, i.e. the detection of a consistent population of cells characterized by the simultaneous presence of CD105, CD71 and ALDH, in the absence of CD34. In this regard, if ALDH^+^/CD34^- ^cell subset may also contain a very rare, primitive stem cell remains to be demonstrated and it is conceivable that a specific marker for the selection of human CD34-negative stem cells needs to be identified before definite conclusions are made, as suggested by other research groups [9,17,18]. However, in our study the majority of cells we identified by ALDH^+^/CD34^-^/CD45^- ^immunophenotype appeared to be committed, lineage antigen positive, non-stem cells. In particular, we demonstrated that these cells display precise erythroid features, *i.e*. CD105 and CD71^bright ^expression in all 23 normal BM samples analyzed. The expression of these two antigens along with CD34 negativity is consistent with erythroid precursors [19,20]. This finding is intriguing because little direct information is available about the phenotypic features of erythroid precursors, as these cells are both sparse and difficult to isolate in sufficient numbers to be studied [19].

In order to further characterize ALDH^+^CD34^- ^cells we performed cell sorting isolation of ALDH^+^CD34^+ ^and ALDH^+^CD34^- ^cell subsets, followed by short term cell culture experiments in which we were able to induce the expression of GPA only on ALDH^+^CD34^- ^cells. These experiments, which strictly followed indications by Malik *et al *[13] who recapitulated the entire erythropoietic process *in vitro*, were the proof of eythroid commitment of ALDH^+^CD34^- ^cells in normal bone marrow.

We also decided to further confirm erythroid commitment of ALDH^+^CD34^- ^cells in a disease model. Interestingly, we demonstrated that three PRCA patients were completely devoid of this cellular subset, thus allowing us to hypothesize the use of this cytometric approach as a diagnostic tool in this kind of disease.

Taken together, our data indicate ALDH as a suitable tool to identify early steps of erythropoiesis as well as to isolate erythroid precursors by cell sorting. Moreover, given the lack of sufficient numbers of reliable erythroid markers in diagnostic panels [15,21] ALDH could also have possible application in the field of PRCA and myelodysplasia FCM diagnosis.

Finally, a possible application of ALDH detection by FCM to the field of acute leukemia may derive from the study of Cheung *et al *[1], in which the authors described ALDH expression in AML. They noted that in AML patients in complete remission, a relevant population of cells characterized by high ALDH activity remained [1]. In this regard, our data concerning multidimensional expression profile of ALDH combined with other hematopoietic antigens in normal BM precursors could represent the basis to distinguish by FCM leukemic from normal ALDH^+ ^cells.

## Conclusion

In this study we demonstrated that ALDH^+^CD34^- ^cells are mainly committed towards erythropoiesis. This finding is new and could be relevant for human physiology studies about erythropoietic differentiation as well as for proposing the utilization of ALDH as a red cell marker in multidimensional flow cytometry characterization of bone marrow from patients with aplastic anemia (mainly PRCA) and myelodysplasia.

## Authors' contributions

PMi and CLP conceived the study, designed the experiments and performed flow cytometry assays. RDN analysed data and wrote the manuscript. PMo and GS prepared cells and performed part of flow cytometry assays. GA, MGo, MR, CP reviewed cytometric files and participated in data interpretation. MGe performed cell sorting experiments. EM participated in study design, data analysis and revision of the manuscript. LDV conceived the study, designed the experiments and wrote the manuscript together with RDN. All authors read and approved the final manuscript.
